# Profiling of Inflammatory Proteins in Plasma of HIV-1-Infected Children Receiving Antiretroviral Therapy

**DOI:** 10.3390/proteomes8030024

**Published:** 2020-09-07

**Authors:** Mahlet Lemma, Stefan Petkov, Yonas Bekele, Beyene Petros, Rawleigh Howe, Francesca Chiodi

**Affiliations:** 1 Department of Microbiology, Tumor and Cell Biology, Biomedicum, Karolinska Institutet, Solnavägen 9, 171 65 Solna, Sweden; mahlet.lemma@ki.se (M.L.); stefan.petkov@ki.se (S.P.); yoni4m@gmail.com (Y.B.); 2 Armauer Hansen Research Institute, P.O. Box 1005, Addis Ababa, Ethiopia; rawleigh.howe@ahri.gov.et; 3 Department of Microbial, Cellular and Molecular Biology, PO Box 1176, Addis Ababa University, Addis Ababa, Ethiopia; abule2002@yahoo.com

**Keywords:** inflammation, protein, profiling, plasma, HIV-1, ART, comorbidities, osteopathology, children

## Abstract

Treatment of HIV-1-infected patients results in improved clinical and immunological conditions, but severe non-AIDS-related conditions still persist. Novel proteomic platforms have identified inflammatory proteins where abundance is dysregulated in adult treated patients, whereas limited data are available in treated HIV-1 infection of children. Using a proteomic plasma profiling approach comprising 92 inflammation-related molecules, we analyzed specimens from 43 vertically HIV-1-infected children receiving antiretroviral treatment (ART) and matched controls in Ethiopia. The infected children were analyzed as a group and separately, according to age of treatment initiation. Proteins displaying a significantly different abundance between groups were hierarchically clustered and presented in heat maps. Random forest analysis was performed to pin-point proteins discriminating between groups; five proteins (STAMBP, CD5, TFG-α, TRANCE, AXIN1) were the strongest prediction factors for treated HIV-1 infection. TRANCE was previously linked to reduced bone mass levels in HIV-1-infected children. CCL4 chemokine, ligand to HIV-1 co-receptor CCR5, was the most critical protein for successful classification between children who initiated ART at different time points. Our data provide evidence that a dysregulated expression of proteins linked to immunological abnormalities and bone metabolism can be found in HIV-1-infected children with prolonged exposure to ART.

## 1. Introduction

In 2015, in order to confine the immunological damage imposed by HIV-1 infection, the WHO recommended antiretroviral therapy (ART) to be initiated in HIV-1-infected children at birth and in adults at diagnosis of HIV-1 infection, possibly already during acute HIV-1 infection. Since the massive ART scale-up availability in low-income countries, the rate of vertical transmission of HIV-1 infection has considerably decreased along with the number of HIV-1-related pediatric deaths. ART administration has a positive impact on a variety of clinical symptoms associated with HIV-1 infection in children [1]. However, ART provided during the chronic phase of HIV-1 infection in children has ameliorated, but not reversed, abnormal features of immune activation [2,3,4].

Observations on long-term ART toxicity in adults suggest that ART can be associated with a greater risk of bone conditions such as fractures and osteoporosis [5,6], renal and metabolic disorders [7,8], cardiovascular diseases [9,10], neuropsychological disorders [11] and liver diseases [12,13]. In HIV-1-infected children, very early ART initiation below 12 weeks of age significantly reduces morbidity and mortality [14]; however, in low-income settings, where difficulties still remain to diagnose HIV-1 infection, the majority of infected infants do not start ART during the first year of life. In fact, although the global HIV statistics presented from UNAIDS showed that 95% of HIV-infected pregnant women in Eastern and Southern Africa had access to ART in 2019 [15], only 58% of children (aged 0–14) living with HIV in this region had access to ART. This delay in ART administration causes multisystem chronic comorbidities of different types than observed in adults [16].

The identification of biomarkers predictive of clinical outcomes and immune reconstitution upon ART administration could lead to improved monitoring of HIV-1-infected patients. In this respect, the use of novel proteomic platforms, by offering the possibility of measuring large numbers of proteins in a small volume of biological sample, may become pivotal in the search of biomarkers during treated HIV-1 infection. Several studies have been conducted to characterize the plasma proteome profiles of HIV-1-infected adults receiving ART. These studies have revealed proteomic signatures associated with statin treatment of HIV-1-infected patients [17], inflammatory markers, metabolic profiles and abnormalities of long-term ART [18,19]. The use of these novel proteomic technologies to pinpoint ameliorated immunological conditions, or toxicology aspects, of ART in HIV-1-infected children has been so far very limited.

In this work, we used a proteomic plasma profiling approach comprising 92 inflammation-related molecules to evaluate HIV-1-associated inflammation and immune activation in 43 HIV-1-infected children receiving ART and matched controls in Ethiopia. The results of this study revealed that long-term ART in HIV-1-infected children is associated with dysregulated expression of several proteins including TRANCE (also named RANKL), previously associated with bone pathology.

## 2. Materials and Methods

### 2.1. Study Participants

HIV-1-infected children (*n* = 43) receiving ART and presenting with undetectable viral load (<40 copies/mL) were recruited to this study along with age-matched healthy controls (*n* = 43). HIV-1 RNA copies were quantified in plasma of HIV-1-infected children using an automated m2000sp Abbott Real-Time HIV-1 assay system following the manufacturer’s protocol (Abbott Laboratories, Abbott Park, IL, USA). Clinical and demographic parameters for the children included in this study are presented in Table 1. The HIV-1-infected children were aviremic and divided into two groups according to the year of sample collection: group 1 comprised 24 HIV-1 children with samples collected in 2018 and group 2 comprised 19 children with specimens collected in 2015. The age-matched control samples were collected in 2018. All HIV-1-infected children were recruited from ALERT Hospital, Zewditu Memorial Hospital and Yekatit 12 Hospital ART clinics and control subjects from Woreda 03 Health Center, Addis Ababa.

Blood samples were collected from all study participants; plasma was isolated and stored at −80 °C Samples were transported frozen to Karolinska Institutet for further analyses. 

### 2.2. Proteomic Plasma Profiling

Plasma samples from 43 HIV-1-infected patients and 43 uninfected controls were analyzed at the Plasma Profiling National Facility, Science for Life Laboratory (Stockholm, Sweden) for the presence of inflammation-related soluble proteins using the Olink Inflammation Panel, which probed for 92 different factors (Olink Bioscience AB, Uppsala, Sweden) [20]. This platform is based on the Proximity Extension Assay technology that involves a pair of oligonucleotide-labeled antibodies (“probes”), which bind to the target protein. A unique PCR target is then formed via a proximity-dependent DNA polymerization event as the probes come in close contact to each other. The new target is finally detected and quantified using qPCR. Quality control of the results showed that more than 90% of the values for IL-20RA, IL-2RB, IL2, FGF-5, IL-22 RA1, NGF-β, IL13, IL-20, IL33 and LIF were below the lower limit of detection and those factors were therefore excluded from the analysis.

### 2.3. Data Analysis

Raw normalized protein expression (NPx) data were transformed by scaling, yielding values with a mean of 0 and standard deviation of 1. Principal component analysis was performed using the scaled values of 82 proteins. NPx values were compared between HIV-1-infected and controls, or between HIV-1-infected patients grouped according to age of ART initiation, using a non-parametric Wilcoxon test. False discovery rate with a significance threshold of 0.01 was used to adjust p values. Significantly different proteins were hierarchically clustered and represented in heatmaps using the *pheatmap* R package [21]. *pROC* was used to calculate the area under the receiver operating characteristic curve (AUC-ROC). Relationship between proteins was computed by Spearman rank correlation and presented in a heatmap. Random forest analysis was conducted using the *randomForest* package. Boxplots and Venn diagrams were created using the *ggplot2* and *VennDiagram* packages, respectively [22,23]. Gene ontology was performed using GOnet [24] analyzing for GO term enrichment with a q value threshold set to <0.01. All other analyses were performed in base R (R Foundation for Statistical Computing, Vienna, Austria) [25]. Unless stated otherwise, a *p* value < 0.05 was considered statistically significant.

### 2.4. Ethical Statement

Ethical clearance to conduct the current study was obtained from the ethical clearance committee of ALERT/AHRI ethics review committee (protocol number P0/06/17) and Ethiopian national ethical review committee (NERC; reference n. 3.10/71/2018). Informed written consent was collected from parents or legal guardians of children in this study. This study was conducted in accordance with the Declaration of Helsinki. The ethical committee at Karolinska Institutet approved the laboratory studies of the collected specimens (approval n 2016/485-32).

## 3. Results

### 3.1. Inflammation-Related Factors in Plasma

We compared the abundance of 82 inflammation-related proteins in plasma of 43 HIV-1-infected children undergoing ART (Table 1) and 43 uninfected controls. The HIV-1-infected children included in this study were all aviremic. 

**Figure 1:**
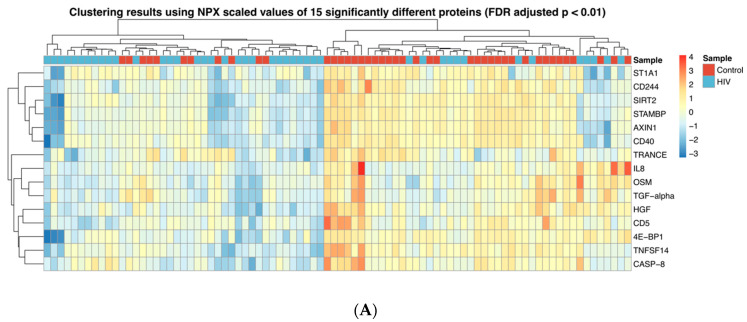

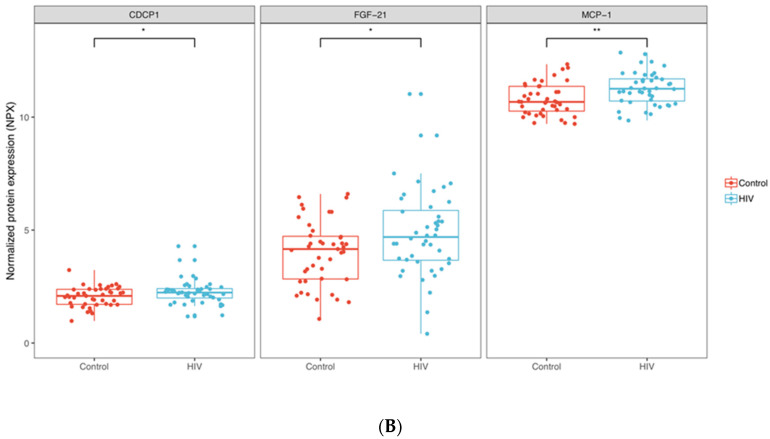
Inflammatory protein profiles in HIV-1-infected children and controls; (**A**) Heatmap showing the expression of 15 proteins where abundance was significantly reduced (*p* < 0.01, FDR adjusted) in HIV-1-infected children compared to controls. (**B**) Box plots showing the abundance of three proteins upregulated in HIV-1-infected children compared to controls. * *p* < 0.05; ** *p* < 0.01.

The abundance of 15 proteins (TRANCE, CD5, IL-8, HGF, TGF-α, OSM, 4E-BP1, ST1A1, AXIN1, STAMBP, SIRT2, TNFSF14, CD40, CASP-8 and CD244) was found to be significantly different between the groups once we selected for proteins with *p* < 0.01 (FDR adjusted); all these 15 proteins were downregulated in the HIV-1 group. Based on these parameters, hierarchical clustering separated the samples in two major groups (Figure 1A). One cluster primarily consisted of 32 samples from uninfected children (out of 45 specimens) and the other was primarily composed of 30 HIV-1-infected samples (out of 41 specimens). The abundance of three proteins, CDCP1, FGF-21 and MCP-1 (*p* < 0.05, not FDR adjusted), was significantly elevated in the HIV-1-infected group compared to the controls (Figure 1B).

**Figure 2:**
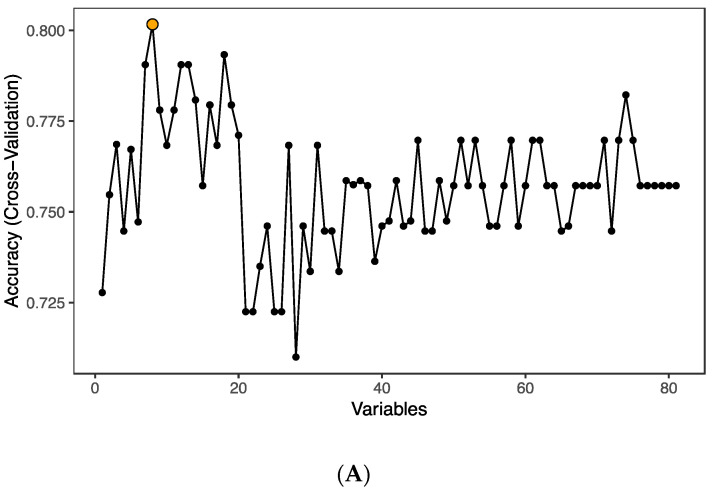

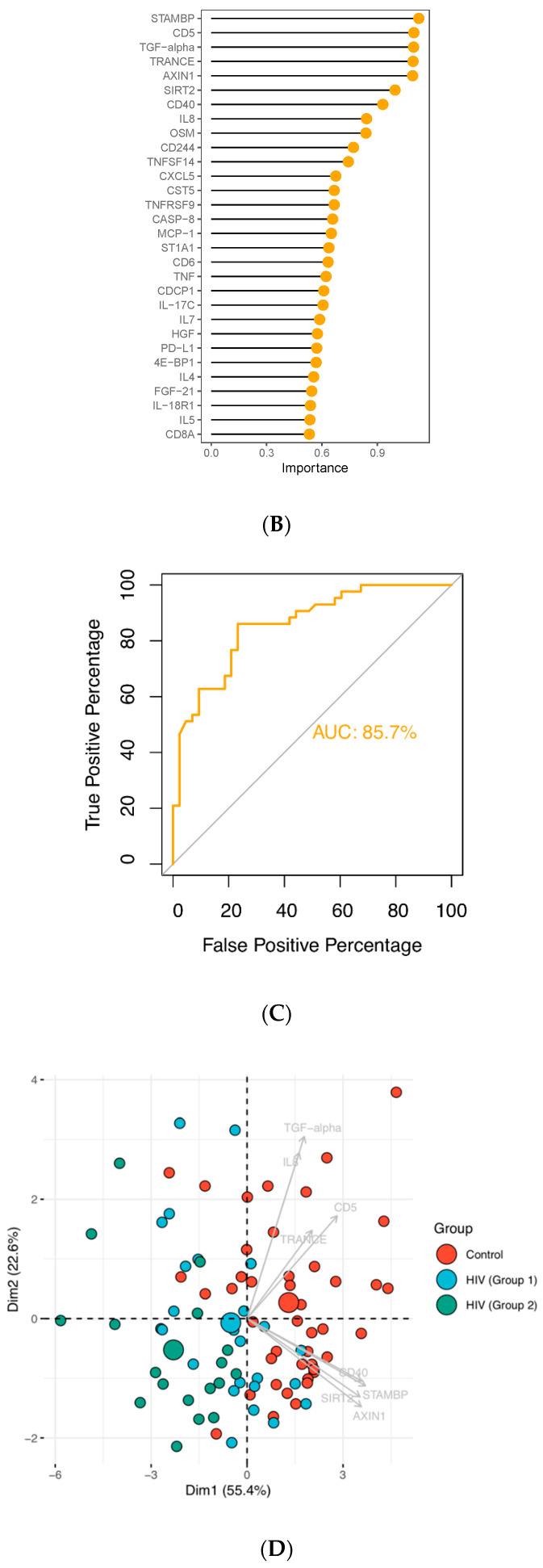
Classification of HIV-1-infected and control individuals using random forest and principal component analysis; (**A**) Optimal number of predictors for HIV-1 status classification. (**B**) Ranking of the top 30 random forest prediction factors based on their importance for distinguishing controls from HIV-1-infected individuals. (**C**) Evaluation of the classification performance by AUC-ROC. (**D**) Principal component analysis based on the eight predictors (TRANCE, STAMBP, AXIN1, CD5, TFG-α, SIRT2, CD40 and IL-8) identified by the random forest algorithm. Large circles represent the mean for each group. Grey arrows represent the principal component loadings.

In order to pin-point proteins discriminating between groups, we performed random forest (RF) analysis using the 82 detected factors on the inflammation panel. The analysis identified eight predictors (TRANCE, STAMBP, AXIN1, CD5, TFG-α, SIRT2, CD40 and IL-8; *p* < 0.05) that could classify samples into groups with an accuracy of 80.2% (Figure 2A); all eight proteins were found to be downmodulated in the infected group. The ranking of random forest prediction factors comprising 82 proteins was performed and the top 30 prediction factors are shown according to their relevance for treated HIV-1 infection (Figure 2B). The classification model performance was formally tested using area under the receiver operating characteristic curve (AUC-ROC), yielding an AUC value of 85.7% (Figure 2C). Principal component analysis (PCA) performed using the RF predictors and subsequent k-means clustering showed that although the individual specimens of the groups failed to form well defined clusters, the mean values of the three groups were spatially distinct with the HIV-1 group 1 positioning closer to the control group (Figure 2D). 

**Figure 3:**
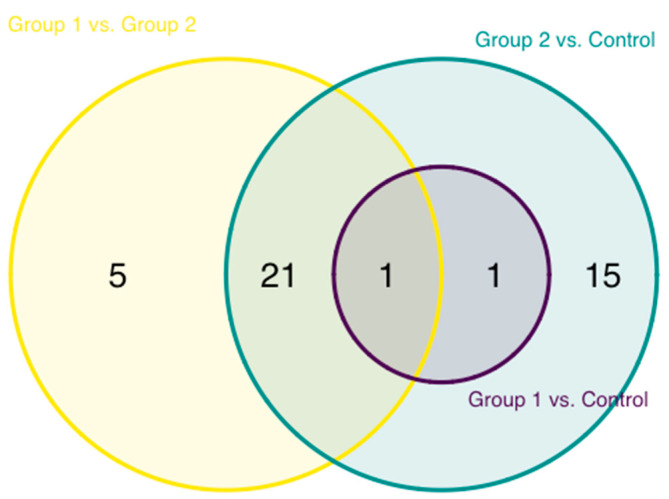
Overlap of the differentially expressed proteins between uninfected controls and the two cohorts of HIV-1-infected children.

A pairwise comparison of the abundance of inflammation proteins of the HIV-1-infected groups and uninfected controls was also performed. The greatest level of difference, 38 proteins, was observed between group 2 and control, followed by group 1 vs. group 2 with 27 proteins and group 1 vs. control, where the abundance of only 2 proteins, TRANCE and STAMBP, was found to be significantly different (Figure 3). These two proteins, in addition, constituted an overlapping difference between both HIV-1-infected and control groups. 

### 3.2. Age at ART Initiation and Inflammation Factors in Plasma

**Figure 4:**
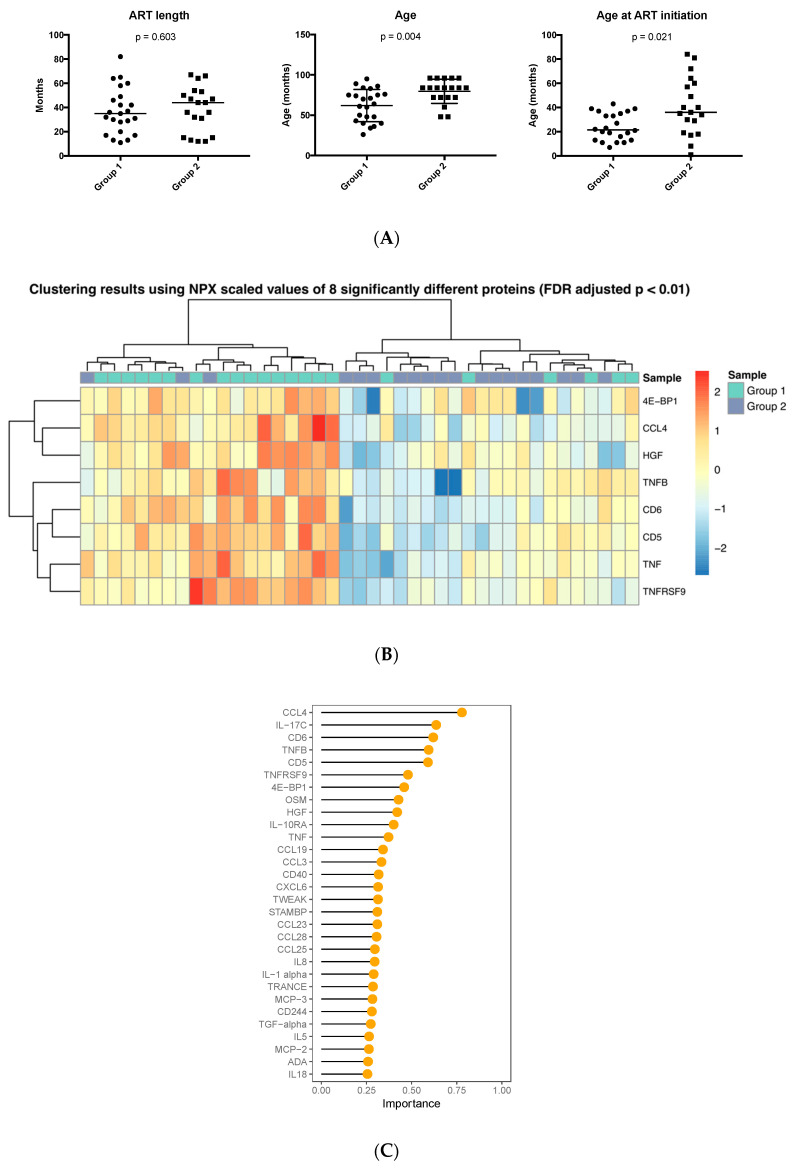
Abundance and ranking of inflammatory proteins according to age of ART initiation in HIV-1-infected children; (**A**) Comparison of ART length, age and age of initiation between the two groups of HIV-1-infected children. (**B**) Heatmap showing the expression of eight significantly different proteins in the two groups of HIV-infected children. (**C**) Importance ranking of the top 30 predictors classifying HIV-1-infected children in the two cohorts.

We next investigated whether the age at which ART was started affected the presence of inflammation-related markers in plasma. The samples in group 1 were obtained from children beginning ART at significantly younger age compared to those in group 2 (median and range 21.5 (7–43) months vs. 36 (1–84) months, *p* = 0.02) whereas the total duration of time under therapy was comparable (Figure 4A). The age of initiation of ART was not available for two patients, which were excluded from the analysis. In addition, at sampling, children included in group 1 had a median age of 60.5 months compared to children in group 2 with a median age of 84 months (*p* = 0.004).

The abundance of eight proteins—4E-BP1, CCL4, HGF, TNFB, CD6, CD5, TNF and TNFRSF9—was significantly different between the two HIV-1-infected groups (*p* < 0.01, FDR adjusted), with all proteins detected at lower abundance in children who started ART at a later time point. Hierarchical clustering based on the eight proteins almost completely coincided with the grouping according to age of ART initiation (Figure 4B). One cluster consisted of 19 samples with 16 of them belonging to group 1 and the other cluster of 22 total samples with 16 pertaining to group 2. RF classification performed with the highest accuracy (78.5%, AUC-ROC = 78.4%) using 35 proteins as predictors of which the 30 most significant are shown (Figure 4C); CCL4 was found to be the most critical protein for successful classification with its abundance higher in the group initiating ART at a younger age. 

### 3.3. Enriched Pathways in HIV-Infected Patients

To better grasp the overall significance of the multiple factors found to be dysregulated during ART, we performed gene ontology (GO) analyses using the factors found to be different between the experimental groups as input gene sets. GO analysis comparing all HIV-1-infected children with healthy controls was performed using the 15 proteins identified as statistically significant (*p* < 0.01, FDR adjusted; Figure 1A). This gene set showed statistically significant association with 61 cell processes (*p* < 0.01, FDR adjusted) of which the top three were “cell surface receptor signaling pathway”, “signal transduction” and “cell response to stimulus.” These process associations covered 80% or more of the input gene list (Table 2).

In addition, we also performed a GO analysis using the proteins that were found in different abundances between patients who began ART at different ages (group 1, early; group 2, late). The input for the analysis consisted of eight genes coding for the proteins in question (Figure 4) and resulted in 22 significant cell process associations. Cell process associations covering more than 80% of the gene list included “cellular response to organic substance” and “signal transduction” (Table 2).

## 4. Discussion

Diagnosis at birth and early ART initiation are essential to prevent progression of disease in vertically HIV-1-infected children; however, a residual low-grade inflammation still persists in treated patients [2]. This study examined 92 inflammation factors in plasma of HIV-1-infected children through a novel proteomic platform; the results showed that HIV-1-infected children despite receiving ART for more than 30 months exhibited several dysregulated inflammatory pathways as compared to non-infected subjects. Three proteins (CDCP1, FGF-21 and MCP-1) were detected where abundance was higher in the HIV-1-infected group compared to the controls and, among these proteins, the monocyte chemoattractant protein 1 (MCP-1) showed the highest abundance difference between the groups. MCP-1, also denominated CCL2, plays the role of attracting monocytes, but also other immune cells, to the site of inflammation. This protein has been recently linked to HIV-1-associated neurocognitive disorders [26] but its role in pediatric HIV-1 infection has not been investigated.

We identified 15 proteins, which had lower plasma abundances in HIV-1-infected children; eight of these proteins including STAMBP, CD5, TFG-α, TRANCE, AXIN1, SIRT2, CD40 and IL-8 were also identified as classification predictors when a random forest classification algorithm was applied and the first five proteins (STAMBP, CD5, TFG-α, TRANCE, and AXIN1) in this group were the strongest prediction factors for treated HIV-1 infection. 

The HIV-1-infected children were grouped in relation to the time point of treatment initiation; the children who initiated ART at a younger age displayed fewer differences in the number of inflammation factors as compared to non-infected controls; the number of inflammatory proteins which differed in abundance compared to controls was, on the other hand, higher in children treated at a later time point. Two proteins, TRANCE and STAMBP, had a reduced abundance in both groups of HIV-1-infected children compared to controls irrespectively of the time of ART initiation.

The involvement of CD5 in modulating signaling through the antigen receptor in both T and B cells has been widely reported [27]. The soluble form of CD5 (sCD5) can be found at low concentrations (pg/mL range) in the serum of healthy individuals, generated through proteolytic cleavage upon lymphocyte activation [28]; sCD5 has been recently demonstrated to exert an immunomodulatory effects on experimental tumor models [29]. Children who started ART at an earlier age had a higher plasma abundance of CD5 in plasma, likely reflecting an improved capacity to respond to immune activation than children treated at a later time point. 

The abundance of the transforming growth factor alpha (TFG-α), produced by keratinocytes, macrophages and brain cells was reduced in HIV-1-infected children. TGF-α through binding to the epidermal growth factor receptor (EGFR) promotes wound healing and tissue repair in the brain [30]. The association of TGF-α with HIV-1 infection has not been extensively studied. The involvement of TGF-α and CXCL-1 with increased telomer length was reported in a study addressing systemic inflammation in a group of HIV-1-infected adults on long-term suppressive ART [18]. TGF-α has been found to be a potent bone-resorbing agent, with a proposed etiologic role in the hypercalcemia of malignancy [31]. 

Osteopathology and reduced levels of bone mass are reported in HIV-1-infected children and adolescents [32], which could be related to the virus itself, or to ART provided either during pregnancy or childhood (reviewed in [33,34]). These pathogenic features are difficult to correct and young HIV-1-infected adults present with a lower peak bone mass and compromised bone strength [34]. It is of interest that among the five most relevant proteins predicting the HIV-1 status and found at lower abundance in HIV-1 children in our study, three are involved in HIV-induced bone loss, RANKL (also denominated as TRANCE), STAMBP and AXIN1. 

The receptor activator of NF-κB (RANK), the ligand RANKL and its physiological inhibitor osteoprotegerin (OPG), produced by the vascular, immune and skeletal systems, participate in bone homeostasis. Dysregulated production of OPG and RANKL from B cells has shown to be involved in osteoclastogenesis and bone loss during HIV-1 infection (reviewed in [35]). In spite of the strong link of the OPG/RANKL-RANK pathways with bone pathology during HIV-1 infection, plasma soluble OPG and RANKL did not reflect changes in bone resorption between HIV-infected and HIV-negative individuals [36]. As found in our study, Babu H and collaborators (2019) also reported a reduced plasmatic level of RANKL in adult ART-treated HIV-1-infected patients compared to non-infected controls; lower level of RANKL in plasma has also been reported to be predictive of non-traumatic fracture [37]. The abundance of OPG, which is also among the molecules measured in the inflammation panel in our study, was not different between the HIV-1-infected children and controls. The STAM binding protein (STAMBP), also denominated as associated molecule with the SH3 domain of STAM (AMSH), potentiates bone morphogenetic protein (BMP) signaling by antagonizing the inhibitory action of SMAD6 and SMAD7 [38]. An additional molecule, AXIN1, among the five associated with the HIV-1 status in our analyses may have relevance for bone dysfunctions in HIV-1 infection. The micro-RNA miR-539 was shown to promote differentiation, proliferation and apoptosis of osteoclasts through the AXIN1-dependent wingless-Int (Wnt) signaling pathway in osteoporotic rats [39]. It would be important to conduct studies specifically addressing the role of AXIN1 in osteoclast apoptosis during treated HIV-1 infection in children. It must be noted that comparable levels of RANKL and STAMBP were found in HIV-1-infected children independently of the time of ART initiation suggesting that the expression of these proteins remains affected in spite of long-term ART or as a result of the therapy.

The early ART strategy in HIV-1-infected children has a beneficial effect on both clinical and immunological parameters linked to HIV-1 infection in children [14,40]. Our cohort consisted of two groups of infected children, who initiated therapy at two distinct time points from birth (21.5 versus 36 months). Even though both groups achieved full viral suppression with no detectable viral load, we showed that children treated early displayed a lower extent of protein dysregulation with a protein profile comparable to what found in control children. We identified CCL4 as the most important proteins discriminating these groups. CCL4, a ligand for the CCR5 chemokine receptor acting as an HIV-coreceptor, is well known as one of the factors produced during primary infection by both CD8^+^ and CD4^+^ T cells that can suppress CCR5-tropic HIV-1 strains [41,42]. It is important that the levels of CCL4 increase in children receiving ART at an earlier age in our cohort of HIV-1-infected children, suggesting improved immunological responses.

The limitations related to the cross-sectional nature of our study should be discussed. In order to confirm the reliability of the identified markers, possibly reflecting pathologies in HIV-1-treated children, the results from this study should be supported by proteomic analyses conducted on longitudinal specimens obtained from HIV-1-infected children receiving ART. One additional weakness of this study is that CD4^+^ T cell counts were not available for all children included in this study and therefore this important parameter, which has pivotal relevance in the context of HIV-1 immunodeficiency, cannot be directly related to our findings on plasma protein profiling. As vertical transmission of HIV-1 in Ethiopia remains a serious problem with a prevalence of transmission higher than 10% in some regions of the country [43,44,45], we propose that the results of the present work, despite its limitations, may serve as a foundation for understanding the effect of ART on HIV-1-infected children committing to a life-long course of treatment.

## 5. Conclusions

Collectively, our data provide evidence that prolonged exposure of HIV-1-infected children to ART, even when successful, is associated with dysregulated expression of proteins linked to neuropathology of HIV-1 infection, immunological abnormalities and bone metabolism. These abnormalities are likely important players in osteopathology reported to occur in HIV-1-infected children [31]. We also show that earlier administration of ART can potentially ameliorate inflammatory protein profiles in treated children, suggesting that ART provided at birth may further improve this picture.

## Figures and Tables

**Table 1:** Clinical characteristics of children included in this study.

Characteristics	Controls (*n* = 43)	HIV-1 Infected (*n* = 43)	
Median age (range) in months	60 (43–84)	Group 1 (*n* = 24)	Group 2 (*n* = 19)
		60.5 (26–95)	84 (48–96)
Gender			
Female	21	14	7
Male	22	10	12
Viral load			
Aviremic	NA	24	19
Viremic	NA	0	0
ART			
ABC + 3TC + LPV/r	NA	8	2
ABC + 3TC + NVP	NA	2	0
AZT + 3TC + NVP	NA	5	15
AZT + 3TC + EFV	NA	4	2
AZT + 3TC + LPV/r	NA	5	0
ART duration (range) in months	NA	33.5 (11–82)	44 (12–67)
Median age (range) in months at ART initiation	NA	21.5 (7–43)	36 (1–84)
WHO stage			
Stage I	NA	23	10
Stage II	NA	1	8
Stage III	NA	0	1
Body mass index	15.4 (12.4–19.8)	14.3 (8.4–20.2)	14.9 (12.9–22.7)

NA = not applicable.

**Table 2:** Ten most significantly associated GO cell processes when comparing HIV-1-infected patients and healthy controls (orange) and children initiating ART at different ages (blue).

	GO Term ID		GO Term	*p*	Adj. *p*	Genes
HIV vs. Control	1	GO:0007166	cell surface receptor signaling pathway	2.90 × 10^−9^	3.23 × 10^−5^	12
	2	GO:0007165	signal transduction	4.83 × 10^−8^	2.66 × 10^−4^	14
	3	GO:0051716	cellular response to stimulus	7.27×10^−8^	2.67 × 10^−4^	15
	4	GO:0023052	signaling	1.17 × 10^−7^	2.78 × 10^−4^	14
	5	GO:0048522	positive regulation of cellular process	1.26 × 10^−7^	2.78 × 10^−4^	14
	6	GO:0007154	cell communication	1.53 × 10^−7^	2.81 × 10^−4^	14
	7	GO:1902533	positive regulation of intracellular signal transduction	1.93 × 10^−7^	3.04 × 10^−4^	8
	8	GO:0048518	positive regulation of biological process	7.67 × 10^−7^	1.06 × 10^−3^	14
	9	GO:0034612	response to tumor necrosis factor	1.02 × 10^−6^	1.25 × 10^−3^	5
	10	GO:1902531	regulation of intracellular signal transduction	1.37 × 10^−6^	1.51 × 10^−3^	9
Group 1 vs. Group 2	1	GO:0002874	regulation of chronic inflammatory response to antigenic stimulus	4.30 × 10^−7^	3.61 × 10^−3^	2
	2	GO:0002678	positive regulation of chronic inflammatory response	8.59 × 10^−7^	3.61 × 10^−3^	2
	3	GO:0071356	cellular response to tumor necrosis factor	1.33 × 10^−6^	3.61 × 10^−3^	4
	4	GO:0002925	positive regulation of humoral immune response mediated by circulating immunoglobulin	1.43 × 10^−6^	3.61 × 10^−3^	2
	5	GO:0034612	response to tumor necrosis factor	1.96 × 10^−6^	3.61 × 10^−3^	4
	6	GO:0019221	cytokine−mediated signaling pathway	2.15 × 10^−6^	3.61 × 10^−3^	5
	7	GO:0071310	cellular response to organic substance	2.39 × 10^−6^	3.61 × 10^−3^	7
	8	GO:0007166	cell surface receptor signaling pathway	2.62 × 10^−6^	3.61 × 10^−3^	7
	9	GO:0002676	regulation of chronic inflammatory response	5.15 × 10^−6^	5.31 × 10^−3^	2
	10	GO:0060693	regulation of branching involved in salivary gland morphogenesis	5.15 × 10^-6^	5.31 × 10^-3^	2
